# Immune Dysregulation, Polyendocrinopathy, Enteropathy, X-Linked Syndrome: A Paradigm of Immunodeficiency with Autoimmunity

**DOI:** 10.3389/fimmu.2012.00211

**Published:** 2012-07-31

**Authors:** Federica Barzaghi, Laura Passerini, Rosa Bacchetta

**Affiliations:** ^1^Division of Regenerative Medicine, Stem Cells and Gene Therapy, San Raffaele Telethon Institute for Gene Therapy, San Raffaele Scientific InstituteMilan, Italy; ^2^Vita Salute San Raffaele UniversityMilan, Italy

**Keywords:** IPEX, FOXP3, Treg, autoimmune enteropathy, neonatal diabetes, neonatal eczema, HSCT

## Abstract

Immune dysregulation, polyendocrinopathy, enteropathy, X-linked (IPEX) syndrome is a rare monogenic primary immunodeficiency (PID) due to mutations of *FOXP3*, a key transcription factor for naturally occurring (n) regulatory T (Treg) cells. The dysfunction of Treg cells is the main pathogenic event leading to the multi-organ autoimmunity that characterizes IPEX syndrome, a paradigm of genetically determined PID with autoimmunity. IPEX has a severe early onset and can become rapidly fatal within the first year of life regardless of the type and site of the mutation. The initial presenting symptoms are severe enteritis and/or type-1 diabetes mellitus, alone or in combination with eczema and elevated serum IgE. Other autoimmune symptoms, such as hypothyroidism, cytopenia, hepatitis, nephropathy, arthritis, and alopecia can develop in patients who survive the initial acute phase. The current therapeutic options for IPEX patients are limited. Supportive and replacement therapies combined with pharmacological immunosuppression are required to control symptoms at onset. However, these procedures can allow only a reduction of the clinical manifestations without a permanent control of the disease. The only known effective cure for IPEX syndrome is hematopoietic stem cell transplantation, but it is always limited by the availability of a suitable donor and the lack of specific guidelines for bone marrow transplant in the context of this disease. This review aims to summarize the clinical histories and genomic mutations of the IPEX patients described in the literature to date. We will focus on the clinical and immunological features that allow differential diagnosis of IPEX syndrome and distinguish it from other PID with autoimmunity. The efficacy of the current therapies will be reviewed, and possible innovative approaches, based on the latest highlights of the pathogenesis to treat this severe primary autoimmune disease of childhood, will be discussed.

## Introduction

Immune dysregulation, polyendocrinopathy, enteropathy, X-linked (IPEX) syndrome is a rare monogenic primary immunodeficiency (PID), characterized by multi-organ autoimmunity. It is caused by mutations in the transcription factor *forkhead box p3* (*FOXP3*), the master gene of T regulatory (Treg) cells. The disease shows an X-linked hereditary pattern: only males are affected, whereas the carrier mothers are healthy.

Although IPEX syndrome is a rare disease, the recent increase in the number of patients referred for diagnosis suggests that the occurrence of the disease has been underestimated so far. At present, 63 *FOXP3* mutations have been published, for an overall number of 136 patients described, and of these about half have been diagnosed in the last 3 years. This also indicates that the awareness of the disease has been growing with a better understanding of the role of FOXP3 and Treg cells in maintaining peripheral tolerance.

Overall, the analysis of cases reported so far (Table 1) confirms the relevance of the three main clinical manifestations and their early onset while highlighting the occurrence of unusual symptoms. The genetic analysis is always required for accurate diagnosis, although other tests such as tissue biopsy and/or autoantibody detection are important, as complementary tools, in the diagnostic process and follow-up.

**Table 1:** **Clinical features, therapy and outcome in reported IPEX patients**.

Reported by	Pt^°^	Mutation	Age at onset	Age at diagnosis	Diarrhea	T1DM	Eczema	Eos (/mm^3^); IgE (IU/mL)	Additional clinical findings	Therapy	Outcome^♦^
Peake et al. (1996), Wildin et al. (2001)	V6^f^, 2^f^	c.1290_1309del_insTGG	6 weeks	Post mortem	+	+	+ Peeling skin	3170; 9999–40,000 μg/L	Anemia, lymphadenopathy, sepsis	TPN, plasma IV	Exitus 10 months
Ferguson et al. (2000), Bennett et al. (2001b), McGinness et al. (2006)	F1-V1^f^, V2^f^, 1^f^	c.1150G>A	1 month	9 years	+	−	+	230–900; 755–3492	Pemphigoid nodularis; bullous pemphigoid; infections, asthma	PD, CSA, dapsone, IgIV, rituximab	Alive 14 years
Ferguson et al. (2000), Bennett et al. (2001b)	F1-IV10^f^	c.1150G>A^#^	2 months	–	− (Vomiting)	−	+	936; 250	Hypothyroidism; infection	na	Exitus 10 months
	F1-V4^f^	c.1150G>A^#^	2 weeks	–	+	+	+	620; 30	Hypogammaglobulinemia, infections, sepsis	IgIV	Exitus 2 years
Ferguson et al. (2000), Bennett et al. (2001b)	F1-V5^f^, V7^f^	c.1150G>A	3 weeks	Post mortem	−	+	+	500; 22	Sepsis	IgIV, CSA	Exitus 12 weeks
Chatila et al. (2000)	3-F1; F2^f^ −14, 15, 27, 28	c.1044 + 4A>G; c.750_752delGGA	3 weeks −3 months	na	5/5	5/5	4/5	na; 4/5 hyper IgE	Autoimmune cytopenia 3/5, food allergy5/5	na	Exitus 1/5, alive 4/5
Levy-Lahad and Wildin (2001), Wildin et al. (2001)	1^f^, F1^f^	c.1189C>T^#^	Birth	–	− (Atonic gut)	−	+	na	Hypotonia, hypothyroidism, thrombocytopenia, peritonitis, cholangitis	–	Exitus 19 days
	2^f^, F1^f^	c.1189C>T	Birth	Post mortem	− (Ileo)	+	−	na	Cachexia, hypotonia, thrombocytopenia, peritonitis	–	Exitus 5 weeks
	3^f^, F1^f^	c.1189C>T^#^	Birth	–	+	+	−	na	Infections, sepsis	Dexamethasone, CSA	
Wildin et al. (2001)	3^na^	c.1113T>G	na	na	+	+	+	na	Anemia	HSCT	Alive, age na
	4^na^	c.1150G>A	na	na	+	+	+	na	Hypothyroidism, thrombocytopenia, sepsis	na	Exitus 4 months
Bennett et al. (2001b), Kobayashi et al. (2011)	F2, 5	c.1293_1294delCT	na	na	+	−	−	na	–	HSCT	Exitus age na
Kobayashi et al. (2001, 2011), Fuchizawa et al. (2007), Otsubo et al. (2011)	1^f^, 1^f^, 3, 1^f^	c.227delT	15 days	na	+	−	−	na; +	Thyroiditis, AHA, tubulonephropathy*	FK506, betamethasone	Alive 19 years
Kobayashi et al. (2001, 2011)	2^f^, 4^f^	c.1087A>G	na	na	+	+	−	na; +	Thyroiditis, tubulonephropathy*, infections, sepsis	na	Exitus 3 years
Baud et al. (2001)	1^f^	c.1113T>G^#^	na	–	+	+	Ichthyosis	na	ITP	na	Exitus 4.5 months
	2^f^	c.1113T>G	1 month	4 months	+	+	Ichthyosis	na; 1750	ITP, AHA, autoimmune neutropenia, cholestatic hepatitis	MPD, FK506, HSCT	Exitus 2 years 7 months
Wildin et al. (2002), McMurchy et al. (2010)	1^f^	c.1040G>A	3 months	13 years	+	+	−	na	Infection (sepsis)	PD, CSA, FK506, HSCT	Exitus 14 years
Wildin et al. (2002)	2	c.1044 + 459A > G	<1 month	na	+	+	+	na	Lymphadenopathy, hepatosplenomegaly eczema, hypothyroidism, AHA, immune neutropenia, infections	Steroids, rituximab, IgIV	Alive 5 years
	3^f^	c.748_750delAAG, c.543C>T	2 months	9 years	+	+	−	na	Arthritis, ITP, hepatomegaly, mild hepatitis, progressive renal insufficiency	Steroids, CSA, FK506, rofecoxib, MTX, rituximab, IgIV, HSCT	Exitus 10 years
Owen et al. (2003)	A-1^f^	c.227delT^#^	2 weeks	–	+	−	+	na	Lymphoid infiltration of the pancreas	na	Exitus 6 weeks
	A-2^f^	c.227delT	3 weeks	na	+	−	+	na	Hepatitis	PDN, AZA, CSA	Alive 10 years
Nieves et al. (2004)	1^na^	c.1150G>A	7 months	9 years	+	+	+	+; 33	Alopecia, longitudinal ridging nails, autoimmune neutropenia, severe anemia, subclinical thyroiditis	PD, CSA, IgIV, G-CSF	Alive 11 years
Tanaka et al. (2005), Fuchizawa et al. (2007), Kobayashi et al. (2011), Otsubo et al. (2011)	1, 4, 1, 3	c.1117T>G	2 months	4 months	+	−	−	na; 2895–7275	–	CSA, PD, IgIV, HSCT	Alive 7 years
Bindl et al. (2005)	1^na^	c.968-20A>C	7 years	10 years	+	−	Dermatitis	na; 17,370	CSA induced chronic interstitial nephritis	Steroids, PD, AZA, CSA, MTX, rapa	Alive 15 years
	2^f^	+	<2 months		+	−	+	na; 3000	–	Steroids, FK506, AZA, rapa	Alive age na
	3^f^	+	<2 months		+	−	+	na; 2000	–	Steroids, FK506, AZA, rapa	Alive age na
Mazzolari et al. (2005)	1^na^	promoter region	4 months	<1 year	+	−	+	na; 763	sepsis	MPD, CSA, HSCT	Alive 2 years 4 months
Bacchetta et al. (2006), Gambineri et al. (2008), McMurchy et al. (2010), Passerini et al. (2011b)	1, 12, 12, 12	c.1117-1118TT>GC	neonatal	3 months	+	+	+	768; 8423	–	MMF, HSCT	Alive 9 years
	2 ^f^, 5^f^, 6^f^, 5^f^, 5^f^	c.543C>T, c.970T>C	neonatal		+	−	+	2780; 374	Allergic asthma	None	Alive 7 years
	3, 2, 1, 2	c.3G>A	Neonatal	<1 years	+	+	+	552; 28,800	Hypothyroidism, lymphadenopathy, hepatosplenomegaly	MPD, CSA, HSCT	Alive 10 years
De Benedetti et al. (2006)	1^f^	c.454 + 4A>G	18 days	22 years	+	−	+	N; 200	Recurrent arthritis, psoriasiform dermatitis, hepatomegaly	PD, MPD, CSA, FK506, infliximab	Alive 22 years
	2	c.323C>T	14 months	7 years	+	−	−	N; 74	Steroid-responsive pneumonia and pericarditis, recurrent arthritis	PDN, PD, AZA	alive 7 years
Myers et al. (2006)	1^na^	c.1-7G>T	1 day	Post mortem	+	^§^	−	na	Hypothyroidism, Infections	na	Exitus 54 days
	2^na^	c.1169G>A	4 days	Post mortem	+	+	+	na	Infections	na	Exitus <2 years
Gavin et al. (2006)	1^na^	c.210_210 + 1GG>AC	na	na	+	+	+	na; high	AHA, ITP	FK506, steroids, TPN	Alive 5 months
	2-p1^f^	c.751_753delGAG	na	na	+	+	+	na; high	Thyroiditis, AHA	Intermittent steroids	Alive 6 years
	2-p2^f^	c.751_753delGAG	na	na	+	+	+	na; high	Thyroiditis	FK506	Alive 9 years
	3^f^	g.-6247_-4859del	na	na	+	-	+	na; high	food allergies	FK506	Alive 4 years
Moudgil et al. (2007), Rao et al. (2007)	1, 2	c.303_304delTT	4 months	6 months	+	+	+	–; 1564	Alopecia, AHA, lymphadenopathy, hypothyroidism, MGN, food allergies, infections	TPN, CSA, PD, rituximab, HSCT	Alive 4 years
Heltzer et al. (2007)	1^na^	c.817-1G>A	birth	Post mortem	+	^§^	Rush	na; 5320	–	FK506	Exitus 79 days
	2^na^	c.1061delC	<2 months	2 years	+	−	+	na; 134	–	NGT, infliximab, illeostomy, mercaptopurine, steroids	Alive 4 years
	3^na^	c.210G>T	2 months	na	+	−	+	na; 6	Recurrent airway infections, ITP, motor delay, hypoglycemic seizures, anemia of chronic diseases, osteopenia, hypogammaglobulinemia	TPN, NGT, Rapa, IgIV	Alive 8 years
Rao et al. (2007)	1^na^	Splice junction Intron 9	na	na	+ Colitis	na	+	na	Food allergies, reactive airways disease, AHA, infections	Imuran, CSA, PD, HSCT	Alive 9 years
	3^na^	c.1271G>A	na	na	+ Colitis	na	+	na	food allergies, AHA, MGN, infections	FK506, MMF, PD, HSCT	Alive 5 years
	4^na^	c.1226A>G	na	na	+ Colitis	na	−	na	AHA	TPN, FK506, rituximab, PD, alemtuzumab, HSCT	Alive 1 years
Torgerson et al. (2007), Halabi-Tawil et al. (2009), Patey-Mariaud de Serre et al. (2009), Moes et al. (2010)	IV.1^f^, 6^f^, 8^f^, 2^na^	g.-6247_-4859del	3 weeks	na	+	−	+	950; >3000	Food allergies, cheilitis, onychodystrophy, recurrent infections, sepsis, Hp gastritis	TPN, FK506, Rapa	Alive 6 years
	IV.2^f^, 7^f^, 7^f^, 1^na^	g.-6247_-4859del	5 weeks	na	+	−	+	2400; 365–>2000	Food allergies, cheilitis, recurrent infections, sepsis	TPN, steroids, FK506, AZA, Rapa	Alive 9 years
Lucas et al. (2007), McLucas et al. (2007)	1^f^	Exon 10^#^	<1 years	6 years	+	−	Dermatitis	na	Hypogammaglobulinemia, anemia, pneumonias, laryngeal papillomatosis, Norwegian scabies	TPN, HSCT	Alive 7 years
Burroughs et al. (2007)	1^na^	c.1271G>A	na	na	+	+	−	na	MGN	HSCT	Alive 6 years
Fuchizawa et al. (2007), Otsubo et al. (2011)	2^f^, 2^f^	c.1150G>A	2 months	na	−	−	+	na	Asthma, Adrenal Insufficiency	Steroids	Alive 10 years
Fuchizawa et al. (2007)	3^f^	c.1150G>A	19 days	na	+	−	+	na	–	–	Alive 15 years
Suzuki et al. (2007)	1^na^	c.1099T>C	8 days	na	+	+	+	na	Liver dysfunction, thrombocytopenia, sepsis	na	Exitus 4 months
Taddio et al. (2007), Gambineri et al. (2008), Passerini et al. (2011b)	1, 11, 11	c.1150G>A	Neonatal	6 years	+	+	+	4900; 1494	Thyroiditis, alopecia, AHA, interstitial pneumonia	Sterois, CSA, FK506, AZA, Rapa, IgIV	Alive 16 years
Lucas et al. (2008)	1^f^	exon 10	3 months	<1 years	+	−	+	5400; na	Thrombocytopenia, Aphthous stomatitis, EBV-induced lymphoma	Rapa, Cx, VCR, PN	Alive, 2 years 6 months
Gambineri et al. (2008)	1	c.2T>C	Neonatal	Post mortem	+	+	−	803; 3910	Sepsis	MPD, CSA, FK506, IgIV	Exitus 3 months
Gambineri et al. (2008), Passerini et al. (2011b)	3, 3	c.210 + 2T>G	Neonatal	6 months	+	+	+	2187; na	Hypothyroidism, hepatitis, sepsis	MPD, CSA, FK506, AZA, IgIV, HSCT	Alive 5 years
Gambineri et al. (2008)	4^na^	c.543C>T	Neonatal	4 months	+	−	−	710; 3	–	MPD, CSA, IgIV	Exitus 5 months
	6^na^	c.816 + 5G>A	neonatal	1 years	+	+	+	na; 517	–	PD, AZA	Exitus 9 months
Gambineri et al. (2008), Passerini et al. (2011b)	7^na^, 7^na^	c.967 + 4A>G	Neonatal	<1 years	+	+	+	700; >2000	Hepatitis	MPD, FK506, AZA	Alive 9 years
Gambineri et al. (2008), Passerini et al. (2011b)	8^na^, 8^na^	c.1015C>G	Neonatal	5 months	+	+	−	na	AIH, AHA, hepatosplenomegaly	MPD, FK506, AZA, Rapa	Exitus 6 months
Gambineri et al. (2008), McMurchy et al. (2010), Passerini et al. (2011a,b)	9, 9, 9	c.1040G>T	Neonatal	1 years	+	+	+	498; 1966	AIH, AIT, anemia, food allergy	PD, CSA	Alive 15 years
Gambineri et al. (2008), McMurchy et al. (2010)	10^na^, 10^na^	c.1040G>T	<1 year	na	Severe chronic gastritis	+	Xerosis	na; >230	Pancreatic exocrine failure, gastrectomy	MPD, CSA	Alive 23 years
Gambineri et al. (2008)	13^na^	c.1121T>G	Neonatal	<1 year	+	−	+	na; 7000	Alopecia, AHA, AIT, CMV infection	MPD, FK506, AZA	Exitus, 11 months
Gambineri et al. (2008), Passerini et al. (2011a,b)	14^na^	c.725T>C	4 months	11 years	+	−	+	1550; 5218	Sepsis, nephropathy	MPD, PD, CSA, FK506	Alive 15 years
Costa-Carvalho et al. (2008)	1^f^	c.1045-3C>G	Birth	na	+	+	+	N; na	Hypothyroidism, AHA, infections	na	Exitus, 11 months
Yong et al. (2008)	1	c.1061delC	2.5 years	<5 years	+	−	Dermatitis	na	Infections	Steroids, mesalazine, infliximab, AZA, 6-MP, rapa	Alive 7 years
	2^f^	c.210G>T	1 week	7 years	+	−	+	na	Respiratory and GI infections, AHA, ITP	IgIV, TPN, steroids, rapa	Alive 8 years
Zhan et al. (2008)	1^na^	c.1139C>T	4 months	5 months	+	−	−	na; high	–	PDN, AZA, FK506, TPN, HSCT	Alive 3 years
Redding et al. (2009)	1^f^	c.1150G>A	6 weeks	na	+	−	+	3753; 157	External otitis, sepsis, bacteremia, AHA	CSA, PDN, HSCT	Alive 2 years
Halabi-Tawil et al. (2009)	1^na^	c.1113T>G	na	na	+	e	+ Erythroderm	na	Congenital ichthyosis, HA, recurrent infections, sepsis	na	na
	2^na^	c.736-1G>A	na	na	+	e	+ Erythroderm	na	Cheilitis, HA, MGN, recurrent infections, sepsis	na	na
	3^na^	c.1101C>G	na	na	+	e	+	na	Recurrent infections, sepsis	na	na
	4^na^	c.560C>T	na	na	+	e	+ Psoriasiform rash	na	Cheilitis, onychodystrophy, HA, recurrent infections	na	na
	5^na^	c.1121T>G	na	na	+	e	+ Psoriasiform rash	na	HA, MGN, recurrent infections, sepsis	na	na
	8^na^	c.751_753delGAG	na	na	+	e	−	na	HA, recurrent infections	na	na
	9^na^	c.751_753delGAG	na	na	+	e	−	na	HA, recurrent infections, sepsis	na	na
D’Hennezel et al. (2009)	1	c.1150G>A	birth	<7 weeks	+	+	Exfoliative dermatitis	na	Hypothyroidism, Respiratory Distress, Seizures, Renal Failure, Pancytopenia	TPN, rapa	Exitus 7 weeks
Patey-Mariaud de Serre et al. (2009)	1^na^	Truncated Protein	1.5 months	na	+	−	Dermatitis	na; N	AIT	na	na
	2^na^	truncated protein	6.5 years	na	+	−	Dermatitis	na; N	Allergic Asthma	na	na
	3^na^	c.1100T>G	1 year	na	+	+	−	na; N	tubulointerstitial nephritis	na	na
	4^na^	p.E251del	4 months	na	+	+	−	na; high	AHA, AIN	na	na
	5^na^	c.1121T>G	2 months	na	+	+	Dermatitis	na; high	AIT, AIN	na	na
	6^na^	c.1113T>G	4 months	na	+	−	Dermatitis	na; N	AHA, AIT	na	na
	9^na^	c.560C>T	11 months	na	+	−	Dermatitis	na; high	AIT, food allergy	na	na
	10^na^	p.E251del	7 months	na	+	+	−	na; high	AIT, AIN, tubulointerstitial nephritis	na	na
	11^na^	Truncated protein	6 months	na	+	+	Dermatitis	na; N	AHA, AIT, MGN	na	na
	12^na^	p.E251del	1 year	na	+	−	−	na; N	–	na	na
Hashimura et al. (2009), Otsubo et al. (2011)	1^f^, 4^f^	c.748_750delAAG	2 months	5 years	− Vomiting	+	+	na; 1141	Food allergy, nephrotic syndrome, infections, AHA, sepsis	CSA, MPD	Alive 5 years
Rubio-Cabezas et al. (2009)	I	c.1222G>A	2 days	na	−	+	−	na, N	Nephrotic syndrome, TIA, chronic diabetes complications	na	Alive 15 years
	IIa^f^	c.1222G>A	3 weeks	na	+	+	−	na; N	Thyroiditis, mucocutaneous candidiasis, infections	na	Alive 12 years
	IIb^f^	c.1222G>A	3.5 months	na	+	+	−	na; N	Thyroiditis, mucocutaneous candidiasis, infections	na	Alive 12 years
	III	c.1010G>A	30 days	na	+	+	−	na; 2266	–	na	Exitus, 13 months
	IV	c.1015C>G	1 week	na	Mal digestion	+	+	na; N	Thyroiditis	na	Exitus, 5.5 months
	V	c.227delT	1 day	na	+	+	−	−; 132	Anemia, neutropenia, thrombocytopenia, dysthyroidism, infections	na	Exitus 8 months
Scaillon et al. (2009), McMurchy et al. (2010)	1^na^	c.1040G>A	8 months	19 years	Gastritis	+	−	N; N	Autoimmune gastritis, pancreatic atrophy, hypo-γ-globulinemia, infections, bronchiectasis,	PDN	Alive 22 years
Dorsey et al. (2009)	1^f^	c.***878A>G	neonatal	4.5 months	+	^§^	+	850; >5000	Sepsis	Rapa, MTX, PD, HSCT	Alive 1 years
Burroughs et al. (2010)	1^na^	c.210 + 2delT	2 months	2 months	+	+	−	1000–2000; 183	Hemolytic anemia, infections	HSCT	Alive, 4 years 9 months
	2^na^	c.816 + 7G>C	na	11 years	+	+	−	2000; 842	Anemia, steroid-dependent interstitial lung disease, membranous nephropathy, hypothyroidism, infections	HSCT	Alive 17 years
Harbuz et al. (2010)	F1^f^ – II3, II4, IV4, IV5, 3, 4, 5, 6	c.816 + 4A>G^#^	na	–	6/6	na	na	na	sepsis	PN	Exitus <5 years 6/6
	F2^f^ – 1	c.816 + 4A>G	2 months	Post mortem	+ Vomiting	−	+	na; >4200	Sepsis	Steroids, TPN	Exitus 3 years
Moes et al. (2010)	3^na^	g.560C> T	Birth	na	+	−	Skin pathol,	High; 5500	Thrombocytopenia, Basedow hyperthyroidism, Hp gastritis, allergy	FK506, HSCT	Exitus 8 years
	4^na^	c.1121T>G	Birth	na	+	−	+	High; 8500	Hemolytic anemia, thrombocytopenia, allergy	FK506, Rapa	Exitus, 14 months
	5^na^	c.751_753delGAG	6 weeks	na	+	−	+	High; 12,500	Hypothyroidism, interstitial nephritis, hemolytic anemia,	FK506, Rapa, HSCT	Exitus 10 years
	6^na^	c.751_753delGAG	4 weeks	na	+	+	Skin pathol	na; 2150	AIH, hemolytic anemia, agranulocytosis	FK506	Exitus 8 months
	7^na^	c.1015C>G	7 days	na	+	+	Skin pathol, no eczema	na; 650	Hemolytic anemia	FK506	Exitus 7 months
Tsuda et al. (2010)	1	c.210 + 1G>A	na	na	+	+	+	na; 3700	Thyroiditis, hepatitis, nephropathy	HSCT	na
	2	c.210 + 1G>A	na	na	−	−	+	na; 3210	nephropathy	na	na
	3	c.543C>T	na	na	+	−	−	na; 1	–	na	na
	4	c.816 + 7G>C	na	na	+	+	+	na; 842	Thyroiditis, nephropathy, recurrent infections	na	na
	5	c.817G>T	na	na	+	−	+	na; 364	Thyroiditis	na	na
	8	c.1150G>A	na	na	+	−	+	na; 2444	–	na	na
	9	c.1157 G>A	na	na	+	−	−	na	–	na	na
	10	c.1169G>A	na	na	+	+	+	na; 2950	Recurrent infections	na	na
	11	c.1190G>A	na	na	+	+	+	na; 657	–	na	na
	12	c.***876A>G	na	na	+	−	+	na	–	na	na
Wang et al. (2010)	1^na^	Intron1	2.5 months	2.5 months	+	+	+	na; +	Thrombocytopenia, hepatitis, hypothyroidism, infections	na	Exitus 4.5 months
An et al. (2011)	1^f^	c.1080_1081insA	20 days	Post mortem	+	+	+	9910; 75	Proteinuria, Sepsis	Supportive treatment	Exitus 1 month
	2^f^	c.1110G>A	14 days	Post mortem	+	−	+	22; 681	Nephrotic syndrome, lymphadenopathy, splenomegaly, pneumonia	Supportive treatment	Exitus 11 months
	3^f^	c.970T>C	26 days	Post mortem	+	+	−	3450; 3	Pneumonia	Supportive treatment	Exitus 5 months
Bae et al. (2011)	1	c.210 + 1G>A	11 months	11 years	+	+	−	N; na	PRCA, MGN, infections	PD	Alive 13 years
Kobayashi et al. (2011)	2	c.1-23G>T	na	na	+	+	−	na	Nephrotic syndrome	CSA, CS	Alive, age na
Otsubo et al. (2011)	5^f^	c.210 + 1G>T	6 months	na	+	+	−	na; na	Nephrotic syndrome	CSA, steroids	Alive 26 years
Kasow et al. (2011)	1^f^	c.1150G>A	1.5 months	<7 months	+	−	+	+; 157–1000	AHA, infections	Rituximab, CSA, PD, HSCT	Alive 3 years 7 months
Lopez et al. (2011)	1	c.748_750delAAG	2 months	na	+	+	+	+; 45	AIH	PD, CSA, AZA, HSCT	Alive 6 years
Passerini et al. (2011b)	17	c.1037T>C	Neonatal	<4 months	+	−	Seborrhoeic dermatitis	467; 1278	Infections, sepsis	MPD, FK506, HSCT	Alive 3 years
	18	c.***876A>G	Neonatal	na	+	−	Seborrhoeic dermatitis	2300; >2000	Hypotonia	TPN, steroids, CSA, HSCT	Alive 8 years
Passerini et al. (2011a)	20	c.816 + 2delT	5 months	27 years	+	−	+	20; 424	AIT, osteomyelitis, arthritis, *S. aureus* sepsis, bronchitis	CSA, MPD, Rapa	Alive 28 years

*Pt, patient; Eos, eosinophils; na, not available; N, within normal ranges; e, unspecified endocrinopathy; ITP, idiopathic thrombocytopenic purpura; AIT, autoimmune thrombocytopenia; AIN, autoimmune neutropenia; PRCA, pure red cells aplasia; MGN, membranous glomerulonephritis; AHA, autoimmune hemolytic anemia; HA, hematological abnormalities (cytopenias, hepatosplenomegaly, or lymphadenopathy); AIH, autoimmune hepatitis; TIA, transient ischemic attack; MSSA, Methicillin-sensitive *Staphylococcus aureus*; PN, parenteral nutrition; TPN, total parenteral nutrition; NGT, nasogastric tube; PD, prednisone; PDN, prednisolone; CSA, cyclosporine; FK506, tacrolimus, MTX, methotrexate; AZA, azathioprine; Cx, cyclophosphamide; VCR, vincristine; HSCT, hematopoietic stem cells transplantation; FU follow-up; TNDM, transient neonatal diabetes; GI, gastrointestinal*.

*In this case, tubulonephropathy could be due both to the underlying disease or to tacrolimus.

*^^°^^Patient ID refers to the enumeration of patients as reported in the original publications listed in column 1*.

*^§^Hypo or hyperglycemia*.

*^f^Positive familial history*.

*^#^The mutation has not been studied in this patient but in other relatives with an IPEX phenotype belonging to the same gender*.

*^♦^The age written in the outcome column refers to the age of the patients at the latest follow-up from each publication*.

IPEX syndrome can be fatal in early infancy if not recognized, therefore a timely diagnosis is essential to start appropriate treatment. Treating IPEX patients poses a threefold challenge: autoimmunity, infections supported by the autoimmune damage, and the severity of the overall picture. Both novel and existing therapeutic approaches will be discussed with an emphasis on the central role of Treg cell impairment in the pathogenesis of IPEX syndrome.

## Genetics of IPEX Syndrome

Immune dysregulation, polyendocrinopathy, enteropathy, X-linked syndrome was described for the first time in 1982 in a large family with 19 affected males across five generations, as an X-linked syndrome with diarrhea that was lethal in most male infants by the first months or years of life (Powell et al., 1982). Only 20 years later, in two unrelated kindred with IPEX phenotype, Chatila et al. (2000) identified mutations in *JM2* (later called *FOXP3*) in the centromeric region of the X chromosome (Xq11.3-q13.3). Shortly after, Bennett et al. (2001b) and Wildin et al. (2001) confirmed that IPEX syndrome is the human equivalent of the *scurfy mouse*, the natural mouse model of the disease, and identified mutations in the *FOXP3* gene in additional IPEX patients. Of note, in the first family described in 1982, the disease mapped to the pericentromeric region of the X chromosome (Bennett et al., 2000), but no identifiable mutation on *FOXP3* was found, so that it was suspected to have a non-coding mutation that affects transcriptional regulation or RNA splicing (Bennett et al., 2001b).

**Figure 1:**
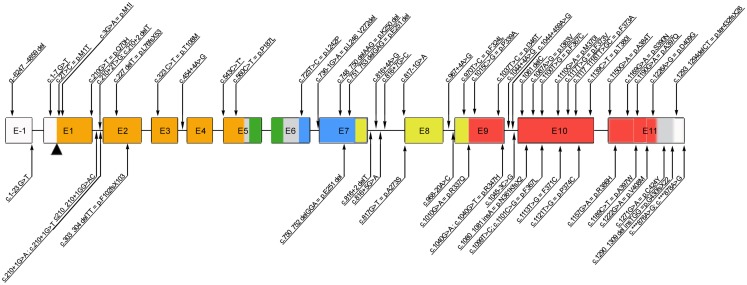
**Schematic representation of the *FOXP3* gene reporting all the mutations published so far**. Annotations refer to both coding sequence and protein, when applicable (www.ncbi.nlm.nih.gov/CCDS, accession number CCDS14323.1). *c543C>T is a polymorphism. E, exon; Color code: orange, N-terminal domain; green, zinc finger domain; blue, leucin-zipper domain; red, forkhead domain.

The highly conserved *FOXP3* gene is composed of 12 exons encoding a protein of 431 amino acids in humans. Among the 63 mutations reported thus far (Figure 1), the majority of them (27/63) alter the C-terminal forkhead (FKH) DNA-binding domain of the protein, while the remaining of the mutations occur outside the FKH domain. The latter include mutations affecting the N-terminal proline-rich (PRR) domain (14/63), the leucine-zipper (LZ) domain (5/63), the LZ-FKH loop (9/63), the region upstream the initial ATG (3/63), and the C-terminal (3/63; Figure 1).

Moreover, mutations of the polyadenylation site of the gene (2/63) have been described, which lead to the expression of an unstable FOXP3 mRNA and usually result in severe, early onset disease (Bennett et al., 2001a; Dorsey et al., 2009; Tsuda et al., 2010; Passerini et al., 2011b). Patients with mutations that abrogate expression of functional FOXP3 protein (i.e., missense or frameshift mutations or splicing defects resulting in a premature stop codon) tend to have severe presentation as well (Gavin et al., 2006; Gambineri et al., 2008; Burroughs et al., 2010; An et al., 2011). Nonetheless, the severity of the disease is not always dependent on the absence of protein expression. The majority of affected individuals have missense mutations (usually point mutations) resulting in a normal or reduced level of expression of mutant protein. Such mutations lead to an impaired transcriptional regulatory activity by altering the binding sites to DNA, the interaction with other molecules (e.g., NFAT, AP1, RORα), or the dimerization of FOXP3 (Figure 1).

Independently from the type or site of the *FOXP3* mutation, all patients described but five (Ferguson et al., 2000; Fuchizawa et al., 2007; Rubio-Cabezas et al., 2009; Scaillon et al., 2009; Tsuda et al., 2010; Otsubo et al., 2011) developed gastrointestinal symptoms (mainly diarrhea). The exact nature of genotype-phenotype correlation has been difficult to pinpoint, especially considering the age at onset and the disease outcome. For example, in 13 patients presenting with the same mutation (c.1150G>A), the onset ranged from birth to 7 months (Table 1). In addition the outcome was influenced by other factors such as timing of the therapeutic intervention, concomitant infections, and each individual patient’s response to therapy.

The histopathological lesions also differ among the patients carrying the same mutation, further suggesting that the genotype does not strictly correlate with phenotypical changes of the target organs (Patey-Mariaud de Serre et al., 2009). This inconsistent correlation between genotype and phenotype may reflect the complex intracellular interactions of FOXP3 (Allan et al., 2005) and also strongly suggests the role of environmental or epigenetic factors that might participate in determining the clinical picture and outcome (Gambineri et al., 2008).

## Clinical Manifestations

Most IPEX patients are born at term after an uneventful pregnancy from unrelated parents. A careful family history may reveal the presence of male subjects in the maternal lineage with similar clinical phenotype, early death, or multiple spontaneous abortions. Notably, these patients may have other affected brothers, but females belonging to the same lineage are usually healthy.

At birth, they may have a normal weight and length without pathological findings. The onset of IPEX syndrome usually occurs in males within their first months of life, but in some cases even after few days or weeks, and can be rapidly fatal if not diagnosed and treated. The most severe cases are characterized by the early onset of a triad of clinical manifestations: intractable diarrhea, type-1 diabetes mellitus (T1DM), and eczema.

*Autoimmune enteropathy* is a hallmark of IPEX syndrome. Patients present with neonatal, watery, and sometimes mucoid or bloody acute diarrhea. This acute severe enteropathy often begins in the first days of life or during breast-feeding, thus showing to be independent from cow milk or gluten introduction in the diet. However, it could be worsen by switching from breast-feeding to regular formula. It typically persists despite dietary exclusions and bowel rest. Since it results in severe malabsorption and significant failure to thrive, parenteral nutrition is often required. In addition to diarrhea, other gastrointestinal manifestations can present, such as vomiting (Ferguson et al., 2000; Hashimura et al., 2009; Harbuz et al., 2010; Otsubo et al., 2011), gastritis (Nieves et al., 2004; Gambineri et al., 2008; Scaillon et al., 2009), ileus (Levy-Lahad and Wildin, 2001), and colitis (Lucas et al., 2007; Otsubo et al., 2011; Table 1).

*Type-1 diabetes mellitus* can precede or follow enteritis. T1DM is present in the majority of patients including newborns, and is usually difficult to control (Peake et al., 1996; Baud et al., 2001; Gambineri et al., 2008). There have been rare cases (6/136) presenting with diabetes mellitus without auto-antibodies (Rubio-Cabezas et al., 2009; Scaillon et al., 2009). Imaging studies or autopsy and histological examination often reveal destruction of the pancreas and intense lymphocytic infiltrate, suggesting that an immune mediated damage of this organ may have a role in the pathogenesis (Wildin et al., 2002; Costa-Carvalho et al., 2008; Rubio-Cabezas et al., 2009).

*Cutaneous manifestations* appear in the first months of life. Similar to diarrhea and diabetes, cutaneous manifestations are very common (95/136) and can be the first sign of the disease (Table 1).

Dermatitis can be eczematiform (mainly atopic dermatitis) (Wildin et al., 2002; Owen et al., 2003; Ruemmele et al., 2008), ichthyosiform (Baud et al., 2001; Rao et al., 2007), psoriasiform (Nieves et al., 2004; De Benedetti et al., 2006), or any combination of the above (e.g., atopic dermatitis and psoriasis coexisting on different areas of the skin) (Halabi-Tawil et al., 2009). Skin involvement is severe and diffuse, characterized by erythematous exudative plaques that could evolve into more lichenfied plaques (Halabi-Tawil et al., 2009). Pruritus can be a major complain in these patients since it is intense and difficult to control with anti-histamine drugs. Cutaneous lesions often show resistance to classic treatments such as topical steroids or tacrolimus and can be complicated by bacterial infections (most commonly *Staphylococcus aureus* and *epidermidis*) with potential development of sepsis (Halabi-Tawil et al., 2009). Other manifestations affecting the integumentary system include: painful and fissurary cheilitis (Halabi-Tawil et al., 2009), onychodystrophy (Halabi-Tawil et al., 2009), and alopecia (Nieves et al., 2004; Moudgil et al., 2007; Gambineri et al., 2008).

Two patients presented with severe *allergies* to food or other allergens causing asthma, skin rashes, and gastrointestinal symptoms in the absence of endocrinopathies. These patients were initially diagnosed and treated as severely allergic individuals (Torgerson et al., 2007). Given this, severe allergic conditions in association with other autoimmune symptoms should raise the suspicion of IPEX syndrome.

The clinical picture can be complicated by the presence of other autoimmune symptoms (Table 1): thyroiditis (27/136) with either hyperthyroidism or, more commonly, hypothyroidism (Kobayashi et al., 2001;Wildin et al., 2001, 2002; Nieves et al., 2004; Myers et al., 2006; Moudgil et al., 2007; Costa-Carvalho et al., 2008; Gambineri et al., 2008; Halabi-Tawil et al., 2009; Rubio-Cabezas et al., 2009; Wang et al., 2010; Otsubo et al., 2011) cytopenias (42/136) such as hemolytic anemia, thrombocytopenia, and neutropenia, and hepatitis (8/136) that may be autoimmune with positive auto-antibodies (Table 1). Renal disease can be related either to autoimmunity or to prolonged administration of nephrotoxic drugs. They are generally described as tubulonephropathy (Kobayashi et al., 2001; Otsubo et al., 2011) and nephrotic syndrome (Gambineri et al., 2008; Rubio-Cabezas et al., 2009; An et al., 2011; Otsubo et al., 2011), although interstitial nephritis (Bindl et al., 2005; Patey-Mariaud de Serre et al., 2009; Moes et al., 2010) and membranous glomerulonephritis (Moudgil et al., 2007; Halabi-Tawil et al., 2009; Burroughs et al., 2010; Bae et al., 2011) have also been found in some patients’ histopathological examinations. A rare manifestation associated with the milder forms of IPEX with delayed diagnosis is arthritis involving one or more joints (Wildin et al., 2002; De Benedetti et al., 2006). Splenomegaly and lymphadenopathy may progress as a result of an ongoing autoimmune lymphoproliferation, as evidenced by the extensive lymphocytic infiltrates in secondary lymphoid organs found in several patients during autopsy (Wildin et al., 2002; Ochs and Torgerson, 2007; Costa-Carvalho et al., 2008). Despite multiple and early autoimmune manifestations typical of IPEX syndrome, it is important to underline that their number may increase with age. IPEX patients’ presentation typically begins early with some of these autoimmune symptoms, and progresses with new manifestations over years.

The clinical spectrum can be worsened by infections, although they are less frequent than the more prominent signs described above. The onset of IPEX syndrome is often associated with infections, however a clear causative role of pathogens in the onset of autoimmunity has not been demonstrated and infections can often be the consequence of multiple immunosuppressive (IS) therapy and poor clinical conditions.

The most frequent infections are pneumonia, airway infections, gastrointestinal, and skin super-infections that may lead to life-threatening sepsis from *Enterococcus* spp. and *Staphylococcus* spp. (Halabi-Tawil et al., 2009). Other common pathogens are *Clostridium difficile*, *Candida albicans*, *Pneumocystis jiroveci*, CMV, and EBV.

## Laboratory Findings

Laboratory tests can be normal at onset. There are no specific diagnostic findings in IPEX syndrome although the laboratory abnormalities consistent with T1DM and severe enteropathy are common. Moreover, other alterations may suggest ongoing autoimmune manifestations in other target organs, such as hypothyroidism, cytopenias, hepatitis, or nephropathy. Markedly elevated IgE levels and eosinophil counts are observed in the majority of patients as an early hallmark of the disease (Table 1). Serum IgA, IgG, and IgM levels are generally normal or low due to the protein-losing enteropathy.

Patients in the acute phase of the disease, prior to IS therapy, can have normal or elevated white blood cell counts. Leukocytosis, if present, is due to an increase in lymphocytes but the percentage of the different lymphocyte subpopulations (CD3, CD4, CD8, CD16, CD19) remains unchanged despite immune dysregulation. The CD4/CD8 ratio is maintained or increased and the T cell repertoire is polyclonal. The percentages of naive and memory T cells are mostly comparable to their age-matched controls. The CD4^+^CD25^+^FOXP3^+^ Treg cells are present (Gavin et al., 2006; Gambineri et al., 2008), but FOXP3 expression can be reduced if *FOXP3* mutation prevents the expression of the protein (Bacchetta et al., 2006) or if the patient is exposed to IS therapy (Gambineri et al., 2008). In addition, *in vitro* proliferative responses to mitogens are normal unless the patient is treated with IS drugs (Bacchetta et al., 2006). The *in vitro* cytokine production shows a decrease in Th1 cytokines and an increase in Th2 (Chatila et al., 2000; Nieves et al., 2004; Bacchetta et al., 2006). The karyotype is normal.

A variety of auto-antibodies are detected in most patients and their presence usually correlates with signs of pathology in the target organs, but their production may also be a sign of immune dysregulation without an obvious pathological linkage (Tsuda et al., 2010).

There is increasing evidence that anti-enterocyte antibodies are characteristic of IPEX patients, although not all patients have been tested because the assay is not widely accessible. The autoimmune enteropathy-related 75 kDa antigen (AIE-75), predominantly expressed in brush border of the small intestine and proximal tubules of the kidney, has been identified as a specific target of the auto-antibodies present in IPEX patients sera (Kobayashi et al., 1998, 1999, 2011; Gambineri et al., 2003; Patey-Mariaud de Serre et al., 2009; Moes et al., 2010).

In addition, a recent study of Kobayashi et al. identified villin, a 95-kDa actin-binding protein, as another brush border antigen aberrantly targeted in IPEX syndrome. Like AIE-75, villin is also expressed both in the microvilli of the small intestine and in the proximal renal tubules. In this study, five out of five IPEX patients showed anti-AIE-75 antibodies and four out of five displayed anti-villin antibodies. None of the control sera from healthy subjects or patients affected by non-IPEX pathologies (e.g., autoimmune enteropathies of different origin, enterocolitis, and colon cancer) were positive for anti-AIE-75 antibodies and only a few were weakly positive for anti-villin antibodies. High levels of anti-villin auto-antibodies have been found only in children with IPEX syndrome (Kobayashi et al., 2011). These findings confirm the specificity of both anti-AIE-75 and anti-villin antibodies for IPEX syndrome. Their link to the tissue damage, the correlation to the progression of the disease, and their predictive value have to be clarified.

Early presence of detectable auto-antibodies against insulin, pancreatic islet cells, or anti-glutamate decarboxylase correlates with occurrence of neonatal T1DM. Moreover, anti-thyroglobulin and anti-microsome peroxidase antibodies are detected in autoimmune thyroiditis even in the absence of functional impairment; Coombs antibodies, anti-platelets antibodies, and anti-neutrophils antibodies are often present in autoimmune cytopenias; anti-smooth muscle (ASMA) and anti-liver-kidney-muscle (anti-LKM) antibodies are positive in autoimmune hepatitis. Recently, Huter et al. (2010) reported that sera from IPEX patients react against keratins, especially keratin 14, suggesting this molecule as a target for autoreactive lymphocytes in the skin of IPEX patients.

Although there is no pathognomonic finding specific to IPEX, biopsies of the affected organs can help in excluding other etiologies. Main histological findings in the gastrointestinal tracts are total or subtotal villous atrophy with mucosal lymphocytic and eosinophil infiltration, but they are not specific for the disease. In a recent work, Patey-Mariaud de Serre and colleagues described the intestinal morphological changes of twelve IPEX patients (Patey-Mariaud de Serre et al., 2009). Three different kinds of lesions were found in the gastrointestinal tract: (1) the graft-versus-host disease-like pattern was the most frequent form observed; (2) the celiac disease-like pattern, found in two patients; (3) depletion of the intestinal goblet cells along with the presence of anti-goblet cell auto-antibodies, reported in one child. Hence, one of these histopathological patterns in the proper clinical context and an association with circulating anti-AIE-75 auto-antibodies would suggest the diagnosis of IPEX syndrome.

In addition, one case reported the autoimmune destruction of pancreatic exocrine cells contributing to the diarrheal disease (Heltzer et al., 2007).

The histopathological changes at the skin biopsies are usually non-specific for IPEX syndrome since there is a wide range of possible dermatological pictures. The clinical and histopathological features of skin pathology of 10 IPEX patients were described by Halabi-Tawil et al. (2009). Either subacute /chronic spongiotic dermatitis or psoriasiform changes, also consistent with a chronic lichenified eczema, have been shown. One out of the four biopsies showed a slight perivascular lymphocytic infiltrate in the upper dermis, while the others showed a moderate to intense superficial dermal infiltrate with the simultaneous presence of eosinophil and lymphocyte infiltrates. Although the majority of skin alterations were compatible with atopic or psoriasiform dermatitis, IPEX patients may present with uncommon allergic (Nieves et al., 2004), autoimmune (Ferguson et al., 2000; McGinness et al., 2006), or infectious (McLucas et al., 2007) dermatological complications.

## Differential Diagnosis

A neonate presenting a single severe manifestation of IPEX syndrome such as enteropathy, diabetes, or newborn erythroderma may pose a diagnostic challenge for the physician. For each of them, the suspicion of IPEX syndrome should be raised once other more common diseases have been excluded.

In a neonate presenting with isolated diarrhea, an autoimmune pathogenesis of the enteropathy is a rare event. Table 2 provides a summary of the possible causes of enteropathy in newborns and infants. IPEX enteropathy, like other diarrheal diseases, may have either an aggressive or insidious onset. When the onset of the diarrhea is acute, microbial origins need to be excluded first. When the diarrhea persists, a wide range of differential diagnosis has to be considered (Murch, 2001). The most common cause is food-sensitive enteropathy, so appropriate exclusion diets should be initiated for an adequate period. Anatomical abnormalities such as malrotation and pseudo-obstruction may cause bacterial overgrowth with chronic diarrhea and malabsorption. If chronic diarrhea is associated to protein-losing enteropathy, lymphangectasia should also be considered. Transport or enzyme disorders induce selective malabsorption of glucose-galactose, lipids, fat-soluble vitamins, amino acids, electrolytes, and zinc (Murch, 2001, 2006). In some of these cases, diarrhea would be abrogated by withdrawing oral feeding. Moreover, malabsorption could be in some cases related to pancreatic disease rather than to an intestinal transport or enzymatic alteration. Nevertheless, the intestinal biopsies in both cases show a normal architecture with intact villous-crypt axis, unlike in IPEX. On the contrary, primary epithelial enteropathies, such as microvillous inclusion disease and tufting enteropathy, are characterized by blunting villi at the intestinal biopsy and usually appear in the first days after birth. They should be excluded if diarrhea is prolonged and continues during total parenteral nutrition (Sherman et al., 2004). Immunodeficiencies, such as severe combined immunodeficiency (SCID) or intermediate forms of combined immunodeficiency (CID), may present first with gastrointestinal symptoms, often fatal in early childhood if untreated (Geha et al., 2007). In the latter cases, diarrhea may be due to a prolonged impairment to clear enteric pathogens or to a primary concomitant autoimmunity. Even metabolic diseases or endocrinopathies could manifest with chronic diarrhea. Further metabolic and hormonal assessment should be considered in such cases. Autoimmune enteropathy is usually a diagnosis of exclusion. Once the aforementioned diseases have been excluded by appropriate clinical or laboratory evaluations, the presence of the following clinical and histological findings indicative of the autoimmune pathogenesis, should be considered: an unresponsiveness to dietary restriction and total parenteral nutrition, an association with other autoimmune conditions (Unsworth and Walker-Smith, 1985), small intestinal villous atrophy with hyperplastic crypt, mononuclear cells infiltrate within the intestinal mucosa (Murch, 1997). Autoimmune enteropathy can be also one of the symptoms of complex forms of immune dysregulation, but other clinical or laboratory features usually help to distinguish them from IPEX syndrome (Table 4).

**Table 2:** **Differential diagnosis of early onset persistent diarrhea**.

Infectious and post-enteritis diarrhea
**FOOD-SENSITIVE ENTEROPATHY OR ENTEROCOLITIS**
Cow’s milk sensitive enteropathy (most frequent)
Celiac disease
Non-celiac gluten sensitivity
Food protein induced enterocolitis
Eosinophilic gastroenteropathy
**ANATOMICAL DEFECTS AND DYSMOTILITY DISORDERS**
Hirschsprung disease
Intestinal lymphangiectasia
Short bowel syndrome (post surgery)
Stagnant loop syndrome (post surgery)
Chronic intestinal pseudo-obstruction
**TRANSPORT DEFECTS**
Chloride-bicarbonate exchanger defect (chloride-losing diarrhea)
Sodium hydrogen exchanger (congenital sodium diarrhea)
Ileal bile acid receptor defect
Sodium-glucose cotransporter defect (glucose-galactose malabsorption)
Abetalipoproteinemia
Hypolipoproteinemia
Acrodermatitis enteropathica (zinc deficiency)
**ENZYMATIC DEFECTS**
Enterokinase deficiency
Disaccharidase congenital defect (lactase, sucrase-isomaltase)
**PANCREATIC MALABSORPTION**
Cystic fibrosis
Shwachman syndrome
**PRIMARY EPITHELIAL CAUSES OF INTRACTABLE DIARRHEA**
Microvillous inclusion disease
Tufting enteropathy
Heparan sulfate deficiency
**IMMUNODEFICIENCIES (USUALLY UNMASKED BY A PATHOGEN)**
Severe combined immunodeficiency (SCID)
Thymic hypoplasia
Class II major histocompatibility (MHCII) deficiency
CD40 ligand deficiency
Neutrophilic specific granule defect
Acquired immunodeficiency syndrome (AIDS)
IBD (very rare in infancy, to be considered as a part of a PID)
**METABOLIC DISEASES**
Mitochondrial myopathy
Wolman disease
**AUTOIMMUNE ENTEROPATHY**

The onset of permanent diabetes mellitus in the neonatal age is described as a rare event (Rubio-Cabezas et al., 2010). Although autoimmune T1DM is diagnosed in over 95% of children presenting with diabetes after 6 months of age (Porter and Barrett, 2004), alternative etiologies should be considered in newborns and young infants presenting with diabetes before 6 months of age (Hattersley et al., 2009). Most of these patients have a monogenic form of disease, even if the responsible gene remains unknown in up to 40% of patients (Edghill et al., 2008). The main monogenic causes of early onset diabetes are mutations in Kir6.2 gene (the inward rectifier subunit of the ATP-sensitive potassium channel of the β cells), in SUR1 gene (the regulatory subunit of the K_ATP_ channel in pancreatic β cells) and in the preproinsulin gene. Mutations of chromosome 6q24 and mutations of the insulin gene may also be considered (Valamparampil et al., 2009; Greeley et al., 2010). The presence of auto-antibodies specific for pancreatic antigens before 6 months of age should however pose the question of *FOXP3* mutation (Greeley et al., 2010). A recent study reported that 4% of male patients with permanent neonatal diabetes were found to have *FOXP3* mutations (Rubio-Cabezas et al., 2009). The diagnosis of IPEX becomes more obvious when diabetes is preceded or followed by other symptoms related to immune dysregulation, such as enteropathy and eczema.

*Skin pathology* is a common finding in infants diagnosed with IPEX syndrome. The absence of other clinical signs may delay the diagnosis, especially in neonates and infants (Nieves et al., 2004). The presentation ranges from mild eczema to severe generalized erythroderma or other unusual skin manifestations with poor response to steroids (Halabi-Tawil et al., 2009; Redding et al., 2009). Focusing on the neonates and infants presenting erythroderma as single diffuse manifestation of IPEX syndrome at onset, Table 3 summarizes the possible clinical pictures that should be considered for differential diagnosis (Hoeger and Harper, 1998; Fraitag and Bodemer, 2010). Erythroderma is an inflammatory skin disorder affecting the majority of the body surface, with subacute or chronic evolution accompanied by scaling skin. In the neonatal period, it can also be the primary manifestation of several conditions. Perinatal or neonatal infections such as Staphylococcal scalded skin syndrome (SSSS) and congenital cutaneous candidiasis may result in diffuse skin involvement. Skin swab and/or skin biopsy is usually diagnostic.

**Table 3:** **Differential diagnosis of erythroderma presenting in the neonatal period**.

**INFECTIONS**
Staphylococcal scalded skin syndrome (SSSS)
Congenital cutaneous candidiasis
**IMMUNODEFICIENCY**
Graft-versus-host disease (GvHD) with underlying SCID
Omenn’s syndrome
**ICHTHYOSES**
Non-syndromic ichthyoses (non-bollous ichthyoses, bollous ichthyoses)
Syndromic ichthyoses (Netherton’s syndrome, Conradi–Hünermann syndrome)
**METABOLIC DISEASES**
Multiple carboxylase deficiencies
Essential fatty acid deficiency
**DRUGS**
Ceftriaxone
Vancomycin
**OTHER SKIN PATHOLOGIES**
Infantile seborrheic dermatitis
Atopic dermatitis
Psoriasis
Cutaneous mastocytosis

Immunodeficiencies may present with extended skin alterations as a result of the immune aggression sustained by autoreactive newborn’s lymphocytes (as in Omenn’s syndrome) or maternal lymphocytes expanding after birth in the immunodeficient host (graft-versus-host disease with underlying SCID). Immunological assessment confirms the diagnosis of PID in these cases (Table 4). If ichthyoses is suspected, skin biopsy is diagnostic. Metabolic disorders can be associated with erythroderma, but usually it is not the only complain and other systemic signs can support the diagnosis. Ceftriaxone or Vancomycin, if recently administered, should be stopped immediately to rule out drug-induced skin reactions. Other common skin pathology of infancy, e.g., atopic eczema and psoriasis, may evolve into erythroderma, but the early presentation, the persistency of the lesions, and the limited response to topical treatment may increase the suspicion of IPEX syndrome. As recently pointed out by Leclerc-Mercier et al. (2010), early skin biopsy has a central role in excluding the majority of these pathological conditions.

**Table 4:** **Differential diagnosis of primary immunodeficiencies (PID) presenting with autoimmunity**.

	IPEX	CD25 def	STAT5b def	OMENN’S	WAS	HIES	ALPS	APECED
Onset	Neonatal, 1 year	<1 year, early infancy	<1 year, infancy	Neonatal, 1 year	1 year, early infancy	Neonatal, 1 year	Neonatal, 1 year	Infancy, young adulthood
Enteropathy	Always	Frequent	Frequent	Frequent	Possible	Not present	Not frequent	Not frequent
Endocrinopathy	T1DM ± thyroiditis	Thyroiditis	GH unresponsiveness	Absent	Absent	Absent	Absent	Hypoparathyroidism, adrenal insufficiency ± T1DM, thyroiditis
Skin lesions	Eczema, erythroderma	Eczema, erythroderma	Eczema	Erythroderma, alopecia	Eczema	Newborn rash, eczema	Urticarial rash	Alopecia, vitiligo
Infections	Rare/secondary to IS	Recurrent/persistent (particularly CMV)	Recurrent (viral), severe varicella	Severe	Frequent	Recurrent pulmonary, cutaneous (*S. aureus*)	Not frequent	Candidiasis
Anemia	Possible	Possible	Rare	Frequent	Possible	Absent	Frequent	Rare
Thrombocytopenia	Possible	Possible	Rare	Possible	Always	Absent	Frequent	Rare
Neutropenia	Possible	Possible	Rare	Rare	Rare	Rare	Frequent	Rare
Others	Failure to thrive, hepatosplenomegaly, lymphoadenopathy, other autoimmune manifestations	Failure to thrive, hepatosplenomegaly, lymphoadenopathy	Growth failure, chronic lung disease, interstitial pneumonia	Failure to thrive, hepatosplenomegaly, lymphoadenopathy, inflammatory pneumonitis and enteritis	Failure to thrive, hemorragies, other autoimmune manifestations, tumors	Characteristic face, cathedral palate, bone fractures	Hepatosplenomegaly, lymphadenopathy, other autoimmune manifestations	Ovarian or testicular failure, gastritis, hepatitis, keratoconjunctivitis
Eosinophilia/hyper IgE	Present	Present	Present	Present	Present	Present	Absent	Absent
Hereditary pattern	X-linked	AR	AR	AR/unknown	X-linked	AD/AR/unknown	AD/unknown	AR
Gene	FOXP3	IL-2RA (CD25)	STAT5b	RAG1/2 (90%), Artemis/IL7RA/, ADA/DNAligase IV/γc/unknown	WASP	STAT3/TYK2/DOCK8/unknown	FAS/FASL/CASP8/CASP10/unknown	AIRE

*WAS, Wiskott–Aldrich syndrome; HIES, hyper IgE syndrome; ALPS, autoimmune lymphoproliferative syndrome; APECED, autoimmune polyendocrinopathy candidiasis ectodermal dystrophy; def, deficiency; IS, immunosuppression; T1DM, type-1 diabetes mellitus; AR, autosomal recessive; AD, autosomal dominant*.

The clinical characteristics that are common in PID with autoimmunity and unique to IPEX are summarized in Table 4. The differential diagnoses with primary immunodeficiencies associated with immune dysregulation and subsequent autoimmune phenomena, such as CD25 deficiency, STAT5b deficiency, Omenn’s syndrome, Wiskott–Aldrich syndrome, Hyper IgE syndrome, autoimmune lymphoproliferative syndrome, autoimmune polyendocrinopathy candidiasis ectodermal dystrophy, should always be considered.

## FOXP3 Dysfunction and Disease Pathogenesis

Forkhead box p3 is a transcription factor, master regulator for the function of thymic-derived regulatory T (nTreg) cells (Wildin et al., 2001; Fontenot et al., 2003; Bacchetta et al., 2007; Gambineri et al., 2008). These cells are among the main subsets of CD4^+^ T cells appointed to maintain peripheral self-tolerance.

CD4^+^CD25^+^FOXP3^+^ T cells can be present in normal percentage in the peripheral blood of the IPEX patients. This was demonstrated not only by immunophenotype, but also by analysis of the Treg-cell-specific-demethylated-region (TSDR; Passerini et al., 2011b; Barzaghi et al., 2012), whose demethylation ensures cell-specific stable expression of FOXP3 (Baron et al., 2007; Wieczorek et al., 2009). Therefore, in IPEX patients *FOXP3*mut Treg cells are physically present but functionally impaired, and this is considered the primary direct cause of autoimmunity in IPEX (Bacchetta et al., 2006; D’Hennezel et al., 2009; Moes et al., 2010). In this respect, IPEX syndrome is the best example of monogenic autoimmune disease due to Treg deficiency. However, autoimmunity in other immunodeficiencies, such as ADA-SCID and WAS, has been recently associated with altered function of Treg cells, regardless of FOXP3 expression (Marangoni et al., 2007; Sauer et al., 2012).

Despite the general consensus on the fact that FOXP3 is fundamental for acquisition and maintenance of suppressive function by nTreg cells (Gavin et al., 2007; Wan and Flavell, 2007; Williams and Rudensky, 2007), it is unclear how the different mutations affect their function. Functional *in vitro* studies on Treg cells of IPEX patients revealed that the degree of functional impairment of the suppressive activity varies among the patients, with complete abrogation of suppressive function in patients with null mutations (Bacchetta et al., 2006). Similarly, mutations in the FKH DNA-binding domain of FOXP3 that caused severe IPEX (p.R347H and p.F373A) were only partially blocked in their ability to reprogram conventional T cells into Treg cells (McMurchy et al., 2010). It may therefore be hypothesized that some mutated forms of the protein retain residual protein activities, thus only partially impairing FOXP3 functions. The molecular mechanisms of Treg-mediated suppression remain controversial, hence our understanding of the impact of different *FOXP3* mutations on Treg cell function is incomplete.

In addition to the well-accepted loss of suppressive function, we recently described that *FOXP3* mutations cause high instability of the Treg cell compartment, with a marked shift to the Th17 cell phenotype of *bona fide* nTreg cells expressing a mutated form of FOXP3 (Passerini et al., 2011b). Indeed, the plasticity between different CD4^+^ T cell subsets is a new and dynamic concept, particularly pronounced between the Th17 and Treg cell compartments (Lee et al., 2009), although the *in vivo* relevance of such phenomenon is controversial (Zhou et al., 2009; Rubtsov et al., 2010). Thus, in addition to the loss of suppressive function, *FOXP3* mutations are associated with inflammation-driven conversion from a regulatory to an effector (i.e., IL-17-producing) phenotype of mutated Treg cells, which may directly contribute to the autoimmune damage in the target organs.

While the necessity of FOXP3 for suppressive function of Treg cells is undisputed, it is unclear whether functional FOXP3 is essential for thymic development of Treg cells in humans. Data from murine models of *FOXP3* deficiency indicate that FOXP3 is dispensable for thymic development of Treg cells, but rather essential for their maintenance in the periphery, as demonstrated in *Foxp3^gfpko^* female mice (Gavin et al., 2007) and in FILIG mice, which display reduced Foxp3 expression in Treg cells (Wan and Flavell, 2007). On the other hand, data from healthy carriers of *FOXP3* mutations and transplanted IPEX patients with low peripheral donor chimerism clearly indicate that only Treg cells expressing a wild type FOXP3 survive long term in the periphery, although leave it unclear whether the selective advantage is already active during thymic differentiation or occurs later on in life (Di Nunzio et al., 2009; Seidel et al., 2009). Our recent observation that *bona fide* Treg cells can be detected by TSDR demethylation analysis in the peripheral blood of IPEX patients both at the onset of the disease and several years after IS treatment, regardless of FOXP3 expression, demonstrates that functional FOXP3 is not necessary for thymic differentiation of Treg cells in humans, as previously demonstrated for murine Treg cells (Gavin et al., 2007), and that FOXP3mut Treg cells can survive and be detected long term, in the peripheral blood of patients with IPEX syndrome (Passerini et al., 2011b; Barzaghi et al., 2012).

Evidences from studies on human and murine models show that Type-1 regulatory T (Tr1) cells can contribute to suppressing the development of autoimmunity in addition to nTreg cells (Roncarolo et al., 2006; Sakaguchi, 2006). We recently demonstrated that Tr1 cells can develop in IPEX patients regardless of FOXP3 expression (Passerini et al., 2011a). This observation suggests that FOXP3-independent immune regulation can potentially contribute to controlling the disease, although Tr1 cells alone do not seem adequate to suppress the initial acute phase of the disease. Thus, it is tempting to conclude that FOXP3 is not necessary for function and development of adaptive Treg cells, the IL-10 producing Tr1 cells.

In humans, FOXP3 is also expressed transiently upon activation, in conventional Teff cells (Allan et al., 2007; Tran et al., 2007; Passerini et al., 2008), in which a still unknown function has been postulated (Ziegler, 2006; McMurchy et al., 2010). This implies that *FOXP3* mutations may also impinge on Teff cell function and suggests that FOXP3-dependent Teff impaired function may directly contribute to the pathogenic mechanism underlying the disease. In support of this hypothesis are the data demonstrating an impaired Th1 cytokine production from IPEX T cells, with relative increase of Th2 cytokines (Chatila et al., 2000; Nieves et al., 2004; Bacchetta et al., 2006). In addition, we observed an increased proportion of IL-17 producing cells in the patients’ PBMC, which could be derived in part from converted Treg, as mentioned above, or in part from Teff cells.

Overall, our current view of the pathogenesis of IPEX syndrome is that, even if impairment of Treg function is the major step, other factors such as inflammation and Th17 elevation can cooperate in maintaining and perpetuating the immune-dysfunction.

## Therapy

Due to the limited and sporadic number of cases reported in literature, it has been difficult up to now to compare different therapeutic strategies and relative outcomes. Therefore, the therapeutic approaches for the treatment of IPEX patients are still based on the experiences in single patients. Moreover, given the unclear genotype-phenotype correlation, the clinical course of the disease and the response to therapy can be variable and not always satisfying. Therapy is therefore targeted to the clinical manifestations and severity of the individual patient. The current treatments available for IPEX syndrome include replacement and supportive therapy, IS therapy, and hematopoietic stem cell transplantation (HSCT). Nutritional support and IS therapy should be promptly started to counteract the initial acute manifestations. A wasting syndrome can acutely affect the outcome of these patients, calling for a collaborative multi-disciplinary effort among clinicians from different specialties such as gastroenterology, infectious disease, and immunohematology.

### Replacement and support therapy

At onset, the patient should be hospitalized and receive a broad-spectrum supportive care (fluids, TPN, albumin) with replacement therapy for endocrine disorders (e.g., insulin and/or thyroid hormones), autoimmune cytopenias (e.g., hemocomponents), or hypogammaglobulinemia (e.g., intravenous immunoglobulins). Prophylactic antibiotics should be used considering the multiple potential sources of infection such as skin lesion, damaged gastrointestinal lining, and central venous catheter. Infectious episodes can drastically exacerbate or complicate the existing clinical symptoms, endangering the patient’s life.

### Immunosuppressive therapy

*Monotherapy or combination immunosuppression* reported so far has shown to be only partially effective in controlling the autoimmune manifestations. Multiple IS therapies are often required to control symptoms (Gambineri et al., 2008).

Glucocorticoids (prednisone and methylprednisolone) are used as the first line therapy to limit progression of organ damage (Gambineri et al., 2008). If the response to prednisone is inadequate, betamethasone (the equivalent oral dose) could have significantly better efficacy (Kobayashi et al., 2001; Taddio et al., 2007). Then other IS drugs can be added onto the steroids regimen. Cyclosporine and/or tacrolimus have been most commonly used in conjunction with steroids (Baud et al., 2001; Wildin et al., 2002; Mazzolari et al., 2005; Taddio et al., 2007; Gambineri et al., 2008). Azathioprine also has been used with steroids and/or tacrolimus with partial control of the disease (Bindl et al., 2005). The ideal dose of medication should be determined to maximize clinical benefit of the individual patient while minimizing side effects.

Thanks to a better understand the disease pathogenesis, clinicians nowadays tend to choose more specific IS drugs, based on the medication’s mechanism of action. Calcineurin inhibitors have partial efficacy with high toxicity and simultaneously suppress Teff cells, expression of FOXP3, and Treg cell function. On the contrary, rapamycin selectively target Teff cells and do not interfere with the function of Treg cells, which are insensitive to mTOR inhibitors (Battaglia et al., 2006; Allan et al., 2008). Even if it is not clear if FOXP3mut Treg cells respond to rapamycin in the same way as FOXP3wt, the use of rapamycin (alone or in combination with azathioprine or steroids) has given promising clinical results in four IPEX cases (Bindl et al., 2005; Gambineri et al., 2008; Yong et al., 2008). In these reports, rapamycin was used not as a first line therapy, but as a second choice when calcineurin inhibitor failed. The dosage used (approximately 0.15 mg/kg/day) was adjusted to maintain serum levels between 8 and 12 ng/mL. In three patients with IPEX syndrome, the combination of rapamycin, methotrexate, and steroid (in one case) and rapamycin, steroid, and azathioprine (in the other two) allowed to obtain clinical remission in all cases and maintain it over time (follow-up of 5 years, 6 months, and 1.5 years, respectively; Bindl et al., 2005). The same positive effect was achieved in one patient with rapamycin and steroid, and with rapamycin monotherapy in another. Both showed clinical remission with a follow-up of 21 and 15 months, respectively (Yong et al., 2008). Based on these positive responses to rapamycin, its use as the first line IS drug in conjunction with steroid might be considered instead of calcineurin inhibitors. Of note, administration of rapamycin should be accompanied by frequent monitoring of serum drug level with appropriate dose adjustment, since the enteropathy may affect the drug intestinal absorption.

In IPEX patients who survived the first years of life, immunosuppression may stabilize the existing symptoms, but flares of the disease may occur and new symptoms may arise despite the therapy.

### Hematopoietic stem cell transplantation

Currently, the only cure for IPEX syndrome is allogeneic HSCT. A summary of the published data regarding HSCT in IPEX patients is provided in Table 5. Early HSCT leads to the best outcome, as the organs are yet to be damaged from autoimmunity and the adverse effects of therapy. For this reason it is fundamental to ensure an early diagnosis. Twenty-eight cases reported received HSCT, 6 out of these 28 patients died despite HSCT or during conditioning (Table 1).

**Table 5:** **Hematopoietic stem cell transplantation in IPEX patients**.

	Baud et al. (2001)	Wildin et al. (2002)		Mazzolari et al. (2005)	Lucas et al. (2007)	Rao et al. (2007)				Zhan et al. (2008)	Seidel et al. (2009)	Dorsey et al. (2009)	Burroughs et al. (2010)		Kasow et al. (2011)
Age at onset	1 month	3 months	2 months	4 months	<1 year	na	2 months^§^	na	na	4 months	neonatal	neonatal	2 months	na	1.5 months
Age at BMT	6 months	13 years	9 years	9 months	6 years	7 years	1 years 5 months	4 years	6 months	5 months	11 months	7 months	9 months	16 years	7 months
Mutation	c.1113 T>G	c.1040 G>A	c.748_750 delAAG	Promoter region	Exon 10	Intron 9	c.303_304 delTT	c.1271 G>A	c.1226 A>G	c.1139 C>T	c.3 G>A	c.***878 A>G	c.210 + 2 delT	c.816 + 7 G>C	c.1150 G>A
Conditioning	ATG, Bu, Cx	Cx, TBI, ATG		Flu, Bu, Cx, ATG	Flu, Bu, ATG	ALM, Flu, Melph				ALM, Flu, Cx	ALM, Flu, Melph	ALM, Flu, Melph	Flu, TBI		ALM, Flu, Thio, Melph
CD34^+^ source	BM	BM	BM	BM	CB	BM	BM	BM	BM	mPB	mPB	BM	mPB	BM	BM
Donor	MRD	MRD	MUD	MRD	MUD, 5/6°	MUD, 8/8°	MUD, 7/8°	MSD, 8/8°	MUD, 8/8°	MUD	MUD, 10/10°	MUD, 10/10°	MUD	MRD	MUD***, 10/10°
Chimerism (%)	100 → 30	100 → 50	100 → 70	70 (Treg 100)	98	na	na	na	na	100	90 Treg, 10 WBC	100	100	100	100 → 30
Remission	Yes*	Yes	Yes**	Yes	Yes	Yes	Yes	Yes	Yes	Yes	Yes	Yes	Yes	Yes	Yes
GvHD	na	No	No	No	Gut 2°	Gut 2°	No	No	No	Skin-gut, 2–3°	Skin 2°	Skin 2°	Skin 2°	Gut 2°	No
Outcome	Exitus	Exitus	Exitus	Alive	Alive	Alive	Alive	Alive	Alive	Alive	Alive	Alive	Alive	Alive	Alive

*na, not available; ATG, anti-thymoglobulin; Bu, busulfan; Cx, cyclophosphamide; TBI, total body irradiation; Flu, fludarabine; ALM, alemtuzumab; Melph, melphalan; Thio, thiotepa; BM, bone marrow; CB, cord blood; mPB, mobilized peripheral blood; MRD, matched related donor; MUD, matched unrelated donor; GvHD, graft-versus-host disease*.

^§^The same case is reported also in Moudgil et al. (2007); °degree of HLA-match; *transient remission; **partial remission; ***T and B depleted.

Among the 15 cases of transplanted IPEX patients reported in detail (Table 5), half of them (8/15) received the transplant before 1 year of age, one of whom died. Among the other half, two patients who received the transplant at 9 and 13 years of age died of infections shortly after. More recently a 16-year-old patient underwent HSCT and a 1-year follow-up was reported. Despite the unfortunate outcome in some patients, the HSCT should be always recommended as the therapy of choice.

Both myeloablative and non-myeloablative conditioning regimens were used in order to limit complications associated with transplantation. The non-myeloablative regimens may enable reduction of both the post-transplant infectious complications and the toxicity of high dose chemotherapy. IPEX patients are very susceptible to the side effects of chemotherapy because of their poor clinical conditions. The use of a non-myeloablative conditioning can more easily result in a partial chimerism.

Both related and unrelated matched donors were used successfully. Only one patient received HSC from cord blood (Lucas et al., 2007) and three from mobilized peripheral blood (Zhan et al., 2008; Seidel et al., 2009; Burroughs et al., 2010), otherwise bone marrow was used as source of HSC (Baud et al., 2001; Wildin et al., 2002; Mazzolari et al., 2005; Rao et al., 2007; Dorsey et al., 2009).

The longest follow-up reported is approximately 8 years post-HSCT for three patients, including one patient transplanted at our Institute (unpublished observations: E. Mazzolari; M. Seidel; R. Bacchetta). Only one of these patients reached full-donor chimerism, however other cases with favorable outcome despite partial chimerism have been described. Therefore, complete donor engraftment in all hematopoietic lineages may not be necessary, but the preferential engraftment of donor Treg cells does indicate that at least the replacement of this cell subset is essential to cure the disease (Seidel et al., 2009). In light of this observation, the choice of drugs for GvHD prophylaxis should aim for the survival of donor Treg cells.

Since wild type Treg cells seem to be sufficient to control the disease, future cell/gene therapy approaches designed to selectively restore the repertoire of Treg cells represent a promising opportunity. Constitutive lenti-viral mediated overexpression of FOXP3 into CD4^+^ T cells can convert Teff into Treg cells both in healthy subject (Allan et al., 2008) and in IPEX patients with different mutations (Passerini, in preparation). When a HLA compatible donor is not available, treatment with engineered T cells could be envisaged. Whether these cells would survive long enough to provide a stable life-long immune regulation without generalized immunosuppression remains to be clarified.

## Conclusion

Immune dysregulation, polyendocrinopathy, enteropathy, X-linked syndrome can be suspected on the basis of clinical and laboratory features, and the timely recognition of the disease leads to significant therapeutic benefits. A multicentre collaborative effort is desirable to implement studies in a wider cohort of patients, in order to achieve a complete knowledge of the disease, to better understand the factors that influence the outcome, and to identify new therapeutic targets. Functional impairment of Treg cells has been recognized as the primary defect at the basis of the immunodeficiency leading to autoimmunity in IPEX syndrome. However, there is evidence that *FOXP3* mutations can contribute to a complex immune-dysfunction, also involving Teff cells, and possibly other cell subsets. Immunological studies on IPEX syndrome have been instrumental in other PID to identify Treg dysfunctions, independent from FOXP3 mutations, as cause of autoimmunity and will most likely advance the knowledge and the therapeutic perspectives of other diseases with immune dysregulation of different origin.

## Conflict of Interest Statement

The authors declare that the research was conducted in the absence of any commercial or financial relationships that could be construed as a potential conflict of interest.
